# Complete Genome Sequence of the Novel Phage MG-B1 Infecting *Bacillus weihenstephanensis*

**DOI:** 10.1128/genomeA.00216-13

**Published:** 2013-06-13

**Authors:** Rodrigo A. F. Redondo, Anne Kupczok, Gertraud Stift, Jonathan P. Bollback

**Affiliations:** Institute of Science and Technology Austria (IST Austria), Klosterneuburg, Austria

## Abstract

Here, we describe a novel virulent bacteriophage that infects *Bacillus weihenstephanensis*, isolated from soil in Austria. It is the first phage to be discovered that infects this species. Here, we present the complete genome sequence of this podovirus.

## GENOME ANNOUNCEMENT

Here, we describe the complete genome sequence of a novel bacteriophage (designated MG-B1) that infects *Bacillus weihenstephanensis*, a psychrotolerant soil-dwelling Gram-positive bacterium belonging to the *Bacillus cereus* group (1). MG-B1 is, to our knowledge, the first phage to be discovered that infects *B. weihenstephanensis*.

Both MG-B1 and its host (*B. weihenstephanensis* MG01) were isolated from secondary growth forest soil in Austria (N 48° 18.929′ E 16° 15.541′). Based on bioinformatics analysis, MG-B1 has podovirus-related genomic features and is more closely related to the Phi29-like group of virulent phages, which are characterized by having a phage-encoded terminal protein (TP) covalently attached at each 5′ DNA end (2). Unlike most other members of this group, MG-B1 appears to be host specific and unable to infect any of the other 16 species of *Bacillus* and related bacteria we tested (*Paenibacillus* spp., *Lysinibacillus* spp., and *Sporosarcina* spp.), including the type specimen *B. weihenstephanensis* DSM11821.

The genome was sequenced using the Ion Shear, Ion OneTouch 200 template kit v2 and Ion Plus fragment library kit (Life Technologies), according to the manufacturer’s instructions. Sequencing was carried out on an Ion Torrent personal genome machine (PGM) platform using the Ion Sequencing 200 kit and Ion Torrent PGM 314 chip (Life Technologies).

Sequence reads were processed on the Ion Torrent server to remove adapter sequences and assign quality scores. Reads were assembled in MIRA 3 (3) and visualized in Tablet 1.12 (4). The assembled genome has a very high coverage (average 71.2×) and average quality calls for the contig of Q73. Direct Sanger sequencing of phage DNA was used to determine the genome ends and resequence low-quality regions. Open reading frames (ORFs) detected with Glimmer (5), ZCURVE_V (6), and Prodigal (7) were combined, and predicted ribosomal binding sites were taken into account for decisions between alternative possible start codons. All predicted ORFs were functionally annotated using BLASTp searches against the nonredundant (nr) database (NCBI) using a maximum E value of 10^-3^, except one identified as homologue to p6 in phage Phi29 (E = 0.027), and at the correct position in the genome.

The genome of MG-B1 is a linear double-stranded DNA (dsDNA) of 27,190 bp, being the largest described so far among the members of the Phi29-like phages. Its inverted short terminal repeats of 22 nucleotides (5′-AAATATAGTGGGGTACACTTTT) are also much larger than those of the previously described species, Phi29, B103, and GA-1, which have 6, 8, and 7 nucleotides, respectively (2, 8). The G+C content is 30.75% and the coding density is 84.41%.

Of the 42 coding sequences (CDSs) detected, 16 had assigned functions, 24 are hypothetical, and 2 are hypothetical conserved. The absolute numbers of start codons found are 38 ATG, 1 GTG, and 3 TTG. Furthermore, 14 of the 16 genes typically found on Phi29-like phages were also found in the corresponding order, including the four main proteins required for replication: p2, p3, p5, and p6 (2, 8). Most of the proteins show similarity with *Bacillus* phages Nf and B103.

### Nucleotide sequence accession number.

The complete genome of bacteriophage MG-B1 is deposited in GenBank under the accession no. KC685370.
